# Comparison of the safety and efficacy of desflurane versus sevoflurane on postoperative cognitive function and early recovery quality in elderly orthopedic patients: a prospective, randomized controlled trial

**DOI:** 10.3389/fmed.2025.1707107

**Published:** 2025-11-26

**Authors:** Ranran Dong, Cunjian Song, Jing Li, Xiangbin Ji, Chunguang Ren, Qingzhi Zhang

**Affiliations:** 1 Department of Anaesthesiology, Liaocheng Infectious Disease Hospital, Liaocheng, China; 2 Department of Anaesthesiology, Shandong Provincial Hospital, Jinan, China

**Keywords:** desflurane, sevoflurane, elderly patients, orthopedic surgery, postoperative cognitive function, early recovery quality

## Abstract

**Objective:**

Desflurane is the most commonly suitable volatile anesthetic for elderly patients due to its low blood solubility, suggesting faster induction and awakening time. This study compared the safety and efficacy of desflurane versus sevoflurane in terms of postoperative cognitive function and early recovery quality in elderly orthopedic patients.

**Methods:**

Eighty elderly patients undergoing orthopedic surgery were included in this prospective, randomized controlled trial. After preoxygenation with a 5 L/min fresh oxygen flow via a facial mask for 5 min, anesthesia was induced using 0.2 μg/kg sufentanil, 1–2 mg/kg etomidate, and 0.2 mg/kg cisatracurium. General anesthesia was maintained through continuous infusion of remifentanil (0.05–0.20 μg/kg/min) and a sevoflurane expiratory concentration of 1–2% or desflurane 2–5%, in combination with air containing 40% oxygen to maintain bispectral index (BIS) values 40–60. Data collected included hemodynamic parameters, time to eye-opening, extubation, following commands, orientation, post-anesthesia care unit (PACU) length of stay, opioid consumption, patient and surgeon satisfaction scores, number of patients willing to repeat surgery with the same anesthesia regimen, and adverse events. Additionally, white blood cell counts, percentages of neutrophils and lymphocytes, and troponin I levels were recorded at baseline and 24 h post-surgery.

**Results:**

The Mini-Mental State Examination (MMSE) scores were lower at 1 h post-surgery in the desflurane group (D group) than in the sevoflurane group (S group), although the difference was not clinically significant (*p* > 0.05). Over 70% of patients in both groups returned to baseline MMSE levels 24 h postoperatively. There were no significant differences in MMSE scores at baseline, 6, 24, or 48 h post-surgery between the groups (*p* > 0.05). Patients in the D group recovered significantly faster, indicated by shorter times to eye-opening, extubation, following commands, and reduced PACU length of stay (*p* < 0.05). Patient and surgeon satisfaction scores and number of patients willing to repeat surgery with the same sedation regimen were significantly higher in the D group (*p* < 0.05), whereas white blood cell counts and percentage of neutrophils were significantly lower 24 h post-surgery in the D group (*p* < 0.05). No significant differences were observed in patient demographics, orientation times, opioid consumption, hemodynamics, adverse events, lymphocyte percentages, or troponin I levels between the groups (*p* > 0.05).

**Conclusion:**

Desflurane was not associated with reduced MMSE scores or postoperative respiratory complications. However, it demonstrated faster recovery times and higher patient satisfaction scores than sevoflurane.

**Clinical trial registration:**

ChiCTR2400093852.

## Introduction

With advancements in medical techniques and economic growth, life expectancy has increased, leading to a rise in the number of patients aged ≥65 years undergoing general anesthesia (1). Elderly patients have a lower basal metabolic rate, diminished digestive function, muscle atrophy, poor adaptive capacity, reduced immunity, and degeneration of vital organs, neurotransmitters, and neurons (2). Consequently, they face an increased risk of postoperative delirium, which occurs in 10–60% of cases despite advancements in surgical and anesthetic techniques (3).

Delirium, a benign temporal disorientation occurring during the transition from anesthesia to wakefulness, typically resolves within minutes or hours. Increasing age is a significant risk factor for cognitive decline and prolonged recovery time from anesthesia (4). Postoperative cognitive decline or dysfunction (POCD), which occurs at least twice as frequently in individuals aged >60 years than in middle-aged or younger groups, is associated with prolonged hospital stays, higher treatment costs, and increased mortality 1 year after surgery (5). Previous studies have suggested that delirium may predict worsening cognitive function within the first year after surgery in patients aged >60 years (6). Additionally, the progressive loss of organ reserve in elderly individuals, coupled with coexisting diseases, can substantially modify pharmacokinetic and pharmacodynamic responses to drugs, significantly affecting the quality of recovery (7). Theoretically, the use of shorter-acting anesthetics and analgesic drugs may reduce postoperative cognitive impairment and confusion in elderly patients. Therefore, selecting appropriate anesthetic drugs is crucial for optimizing postoperative recovery in this population.

The use of volatile anesthetics that are rapidly eliminated with minimal metabolic breakdown may help reduce postoperative delirium and cognitive dysfunction in elderly surgical patients (8). Sevoflurane and desflurane are among the most commonly used volatile anesthetics for maintaining anesthesia. Both agents have low blood-gas partition coefficients (0.42 vs. 0.65), allowing for shorter induction times, rapid emergence, and early recovery of airway reflexes than other soluble inhaled anesthetics (9). Recent studies have indicated that desflurane has the lowest blood solubility, resulting in the fastest induction and awakening. Furthermore, volatile anesthetics may be advantageous for elderly patients with obstructive respiratory dysfunction due to their bronchodilating properties (10). This prospective, randomized controlled trial was designed to compare the safety and efficacy of desflurane versus sevoflurane in terms of postoperative cognitive function and early recovery quality in elderly orthopedic patients.

## Methods

### Study design

This study was a randomized, parallel, double-blind controlled trial. Informed consent was obtained in writing from all participating patients and their legal representatives. The study was conducted between May and September 2024 and was approved by the Ethics Committee of The Liaocheng Infectious Disease Hospital. It was also registered with the Chinese Clinical Trial Registry (ChiCTR2400093852) and adhered to the Helsinki Declaration.

The inclusion criteria were patients aged between 65 and 75 years undergoing orthopedic surgery. Patients were excluded if they had clinically significant cardiovascular, respiratory, hepatic, renal, neurological, psychiatric, or metabolic diseases; a Mini-Mental State Examination (MMSE) score ≤24; American Society of Anesthesiologists (ASA) grades >III; a history of long-term use of narcotic analgesics, sedatives, antidepressants, or alcohol; or if they had participated in other clinical trials within the last 3 months. Patients with body weight exceeding 150% of their ideal body weight were also excluded (11).

An independent investigator generated a random assignment sequence using randomization software and assigned 80 patients to one of two groups. To ensure consistency, the same research assistant—trained in the use of the Confusion Assessment Method (CAM) and MMSE—evaluated all patients and performed chart reviews. While the anesthesiologist could not be blinded due to differences in administration techniques for the two volatile anesthetics, the surgeons, nurses, patients, and other investigators remained blinded to group assignments until the study was completed.

### Anesthesia

No perioperative sedation was administered. Upon arrival in the operating room, a venous catheter was inserted, and lactated Ringer’s solution was infused at 5 mL/kg before anesthesia and then adjusted to 5 mL/kg/h during surgery. Routine monitoring included peripheral oxygen saturation (SpO_2_), electrocardiography (ECG), noninvasive blood pressure, and bispectral index (BIS, E-8000, Beijing Thinking High Medical Technology Co., Ltd., China). End-tidal carbon dioxide tension (PetCO_2_) and concentrations of desflurane and sevoflurane were measured using a gas analyzer (Carestation 650 A1, 3,030 Ohmeda Dr. Madison, USA). After preoxygenation at a fresh oxygen flow of 5 L/min via facial mask for 5 min, anesthesia was induced with 0.2 μg/kg sufentanil, 0.1–0.2 mg/kg etomidate, and 0.2 mg/kg cisatracurium. General anesthesia was maintained using continuous infusion of remifentanil (0.05–0.20 μg/kg/min) and sevoflurane (1–2% expiratory concentration) or desflurane (2–5%) to maintain BIS levels 40–60. Mechanical ventilation was initiated with an inspired oxygen concentration of 40%, a fresh gas flow rate of 2 L/min, a tidal volume of 6–8 mL/kg, a respiratory rate of 12 breaths/min, and a positive end-expiratory pressure of 5 cmH_2_O (if there were no contraindications) to maintain PetCO_2_ between 35 and 40 mmHg. IV boluses of sufentanil and cisatracurium were administered at the discretion of the attending anesthesiologist. At the end of surgery, residual neuromuscular blockade was reversed using 0.02 mg/kg atropine and 0.05 mg/kg neostigmine. Extubation was performed when the following criteria were met: respiratory rate ≥8/min, tidal volume ≥5 mL/kg, hemodynamic stability, recovery of cough reflex, and SpO_2_ ≥ 95% (12). All patients were transferred to the post-anesthesia care unit (PACU) with oxygen flow at 5 L/min and were returned to the ward if the modified Aldrete score was ≥9.

### Outcomes

The primary safety outcome was cognitive impairment measured using the MMSE (a scale from 0 to 30, with lower scores indicating worse impairment) at baseline and at 1, 6, 24, and 48 h post-surgery. A decrease in MMSE >2 points was considered clinically significant (13). Other safety outcomes included PACU length of stay; patient and surgeon satisfaction scores (1 = dissatisfied, 5 = satisfied); adverse events (e.g., respiratory infection, respiratory failure, pleural effusion, atelectasis, pneumothorax, bronchospasm, aspiration pneumonitis); and the number of patients willing to repeat surgery with the same sedation regimen. The incidence of postoperative delirium was assessed using CAM (assesses four criteria: acute onset/fluctuating course, inattention, disorganized thinking, and altered level of consciousness) at 6 and 24 h post-surgery. Delirium is considered present when both the first two criteria and either the third or fourth criteria are present at any of the postoperative assessment points (14). The efficacy outcomes were time to eye-opening, extubation, following commands, and orientation; hemodynamic measurements recorded at various time points (T0: arrival at the operating room; T1: before anesthesia induction; T2: before surgery; T3: 5 min after surgery start; T4: 10 min after surgery start; T5: surgery end; T6: arrival in PACU; T7: 5 min in PACU; T8: 10 min in PACU; T9: pre-discharge from PACU); opioid consumption. Additional measures included white blood cell counts, percentages of neutrophils and lymphocytes, and troponin I levels recorded at baseline and 24 h post-surgery.

### Statistical analysis

Sample size calculations, based on an intergroup difference of our pre-experiment in MMSE decrease at 1 h post-surgery (25.22 ± 1.74 vs. 26.31 ± 1.39, power = 0.8, *α* = 0.05), revealed a required sample size of 36 patients per group. To account for a 10% dropout rate, 80 patients were recruited.

Statistical analyses were performed using GraphPad Prism 8.0 and SPSS 24.0 (SPSS, Chicago, IL, USA). The Shapiro–Wilk test assessed normality, while Levene’s test evaluated homogeneity of variances. Continuous data were presented as means ± standard deviations or medians and interquartile ranges and analyzed using the Student’s *t*-test or Mann–Whitney *U*-test. Repeated measures analysis of variance assessed hemodynamic variables, MMSE, and CAM scores. Qualitative data were reported as numbers and frequencies and analyzed using *χ*^2^ or Fisher’s exact tests. A *p*-value <0.05 was considered statistically significant.

## Results

### Patient demographic characteristics

**Figure 1:**
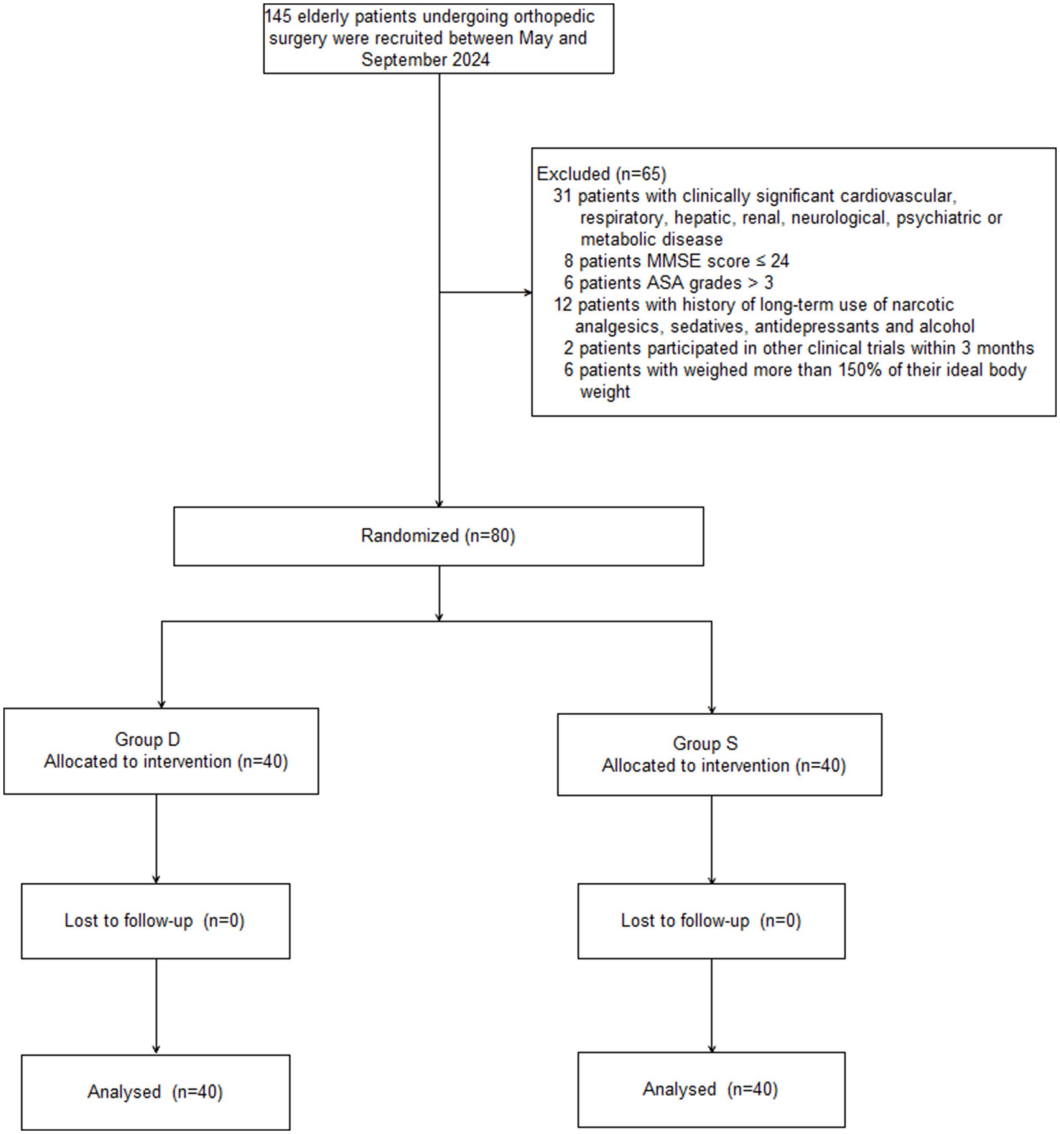
Patient flowchart with CONSORT guidelines.

A total of 145 elderly patients undergoing orthopedic surgery were recruited between May and September 2024. Sixty-five patients were excluded for the following reasons: clinically significant cardiovascular, respiratory, hepatic, renal, neurological, psychiatric, or metabolic disease (*n* = 31); MMSE score ≤24 (*n* = 8); ASA grade >III (*n* = 6); history of long-term use of narcotic analgesics, sedatives, antidepressants, or alcohol (*n* = 12); participation in other clinical trials within the past 3 months (*n* = 2); or weight exceeding 150% of their ideal body weight (*n* = 6). As a result, 80 patients were equally divided into the D and S groups (*n* = 40 each; Figure 1). There were no significant differences in patient demographic characteristics between the two groups (*p* > 0.05; Table 1).

**Table 1:** Demographics and baseline data between the two groups.

Variable	Group D (*n* = 40)	Group S (*n* = 40)	*P*-value
Age (years)	68.98 ± 2.93	68.95 ± 2.61	0.968
Gender (male/female) History of smoking, *n* (%)	24/16 17 (42.50%)	23/17 25 (62.50%)	0.820 0.073
Education (primary school/middle school/high school and above)	13/19/8	15/19/6	0.807
BMI (kg/m^2^)	25.67 ± 1.87	25.39 ± 1.81	0.491
ASA I/II/III (*n*)	10/23/7	7/23/10	0.589
Comorbidity, *n* (%)			
Hypertension	17 (42.50%)	22 (55.00%)	0.962
Diabetes	15 (37.50%)	20 (50.00%)	
Coronary heart disease	15 (37.50%)	22 (55.00%)	

Variables presented as mean ± SD or number of patients *n* (%). BMI, Body Mass Index; ASA, American Society of Anesthesiology.

### Primary outcome

The MMSE scores were lower in the D group 1 h post-surgery than those in the S group, although the difference did not reach clinical significance (*p* > 0.05; Table 2). A transient decline in MMSE was observed in 75% of patients in the D group and 67.5% in the S group 1 h post-surgery. More than 70% of patients in both groups returned to baseline MMSE levels by 24 h postoperatively. However, there were no significant differences in MMSE scores between the two groups at baseline or at 6, 24, and 48 h after surgery (*p* > 0.05; Table 2).

**Table 2:** Comparison of MMSE between the two groups.

Variable	Group D (*n* = 40)	Group S (*n* = 40)	*P*-value
Baseline	27.03 ± 1.14	26.88 ± 1.14	0.558
1 h	25.75 ± 0.84	26.00 ± 0.85	0.059
6 h	26.68 ± 0.92	26.55 ± 0.88	0.535
24 h	26.88 ± 1.02	26.70 ± 0.85	0.407
48 h	27.15 ± 1.03	27.05 ± 1.04	0.666

Variables presented as mean ± SD.

### Other outcomes

**Figure 2:**
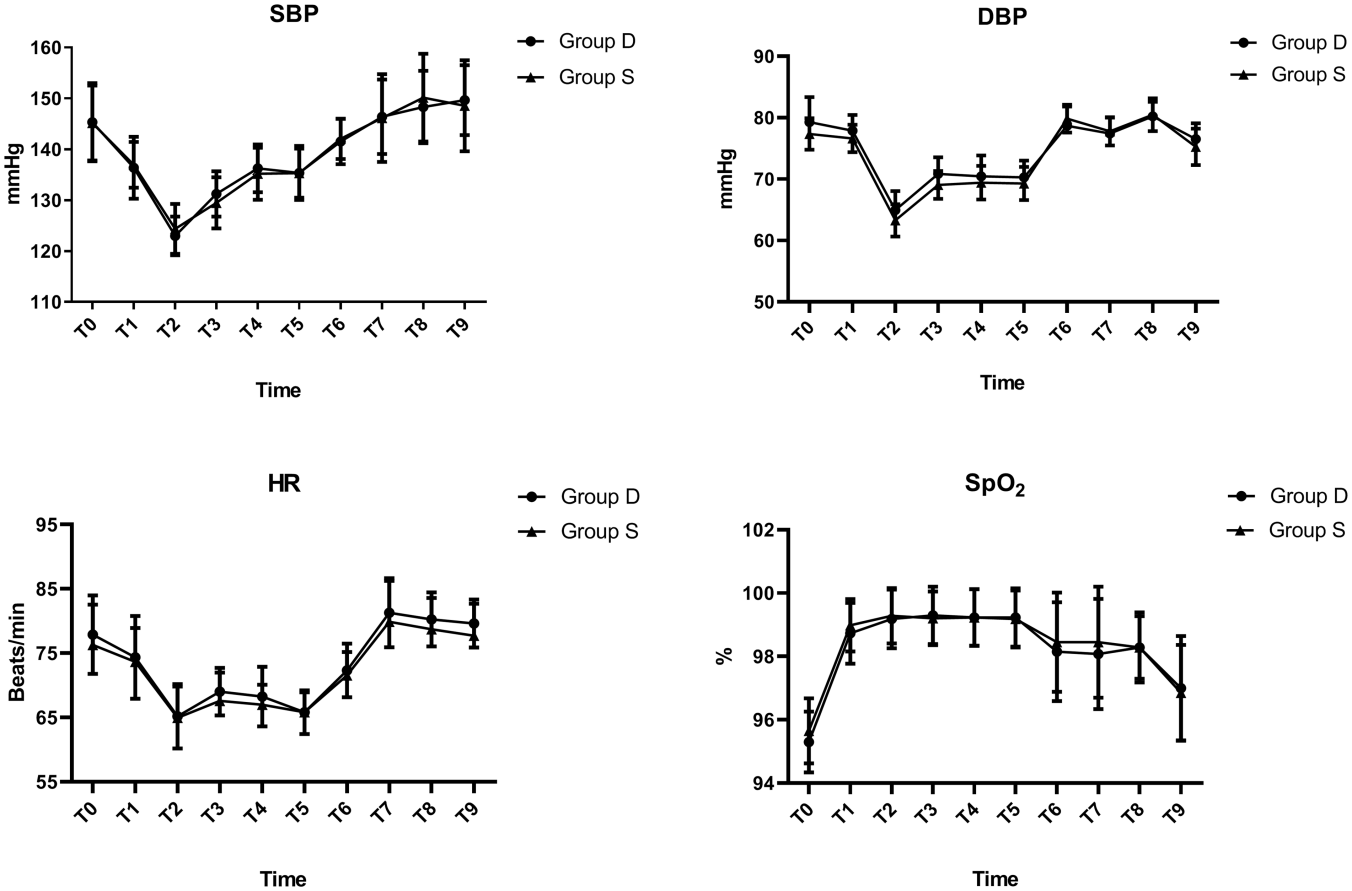
Hemodynamic changes between the two groups.

Patients in the D group recovered significantly faster in terms of time to eye-opening, extubation, following commands, and length of stay in the PACU (*p* < 0.05; Table 3). However, no differences were observed between the two groups in time to orientation, perioperative opioid consumption, or hemodynamic variables (*p* > 0.05; Table 3, Figure 2). Compared with the S group, patients in the D group reported significantly higher satisfaction scores and a greater willingness to repeat surgery with the same sedation regimen (*p* < 0.05; Table 3).

**Table 3:** Intra- and postoperative parameters between the two groups.

Variable	Group D (*n* = 40)	Group S (*n* = 40)	*P*-value
Time metrics			
Time to open eyes (min)	4.95 ± 1.48	7.08 ± 1.25^*^	0.001
Time to extubation (min)	7.15 ± 1.10	9.13 ± 1.22^*^	0.001
Time to orientation(min)	10.13 ± 1.29	10.50 ± 1.24	0.188
Time to follow commands (min)	10.80 ± 1.16	11.45 ± 1.18^*^	0.015
Length of stay in the PACU (min)	17.20 ± 1.57	18.23 ± 1.53^*^	0.004
Consumption of sufentanil (μg)	16.39 ± 2.32	16.35 ± 2.14	0.936
Consumption of remifentanil (μg)	547.07 ± 92.82	573.25 ± 88.40	0.200
Consumption of etomidate (mg)	11.03 ± 2.15	10.65 ± 2.32	0.440
Consumption of cisatracurium (mg)	14.60 ± 1.13	14.49 ± 1.18	0.686
Consumption of desflurane (MAC)	3.86 ± 0.50	—	
Consumption of sevoflurane (MAC)	—	1.69 ± 0.85	
Operative time (min)	103.98 ± 11.72	97.38 ± 18.02	0.056
Anesthesia time (min)	123.80 ± 10.82	120.18 ± 11.46	0.150
Patient satisfaction scores	4.25 (4.00–4.75)	4.00 (3.75–4.75)^*^	0.034
Surgeon satisfaction scores	4.50 (4.00–4.75)	4.25 (3.75–4.75)	0.224
Willing to the repeat surgery with the same sedation regimen, *n* (%)	36 (90.00%)	27 (67.50%)^*^	0.014

Variables presented as mean ± SD, median (interquartile range) or number of patients *n* (%). MAC, minimum alveolar concentration; PACU, Post Anesthesia Care Unit. *p < 0.05 vs. Group D.

There were no significant differences between the two groups in the incidence of postoperative respiratory complications, emergence cough, nausea and vomiting, or postoperative delirium (*p* > 0.05; Table 4). All observed adverse events were of minor severity (*p* > 0.05; Table 4).

**Table 4:** Adverse events between the two groups.

Variable	Group D (*n* = 40)	Group S (*n* = 40)	*P*-value
Respiratory complications			
Respiratory infection	6 (15.00%)	6 (15.00%)	1.000
Respiratory failure	2 (5.00%)	2 (5.00%)	1.000
Pleural effusion	4 (10.00%)	8 (20.00%)	0.210
Atelectasis	4 (10.00%)	5 (12.50%)	1.000
The incidence of postoperative delirium			
6 h	4 (10.00%)	4 (10.00%)	1.000
24 h	1 (2.50%)	1 (2.50%)	1.000
The incidence of nausea and vomiting	5 (12.50%)	10 (25.00%)	0.152
The incidence of emergence cough	4 (10.00%)	5 (12.50%)	1.000
Severity of adverse events			
Grade 1	25 (62.50%)	34 (85.00%)	
Grade 2	1 (2.50%)	2 (5.00%)	1.000
Grade 3	0	0	
Grade 4	0	0	

Variables presented as number of patients *n* (%).

**Figure 3:**
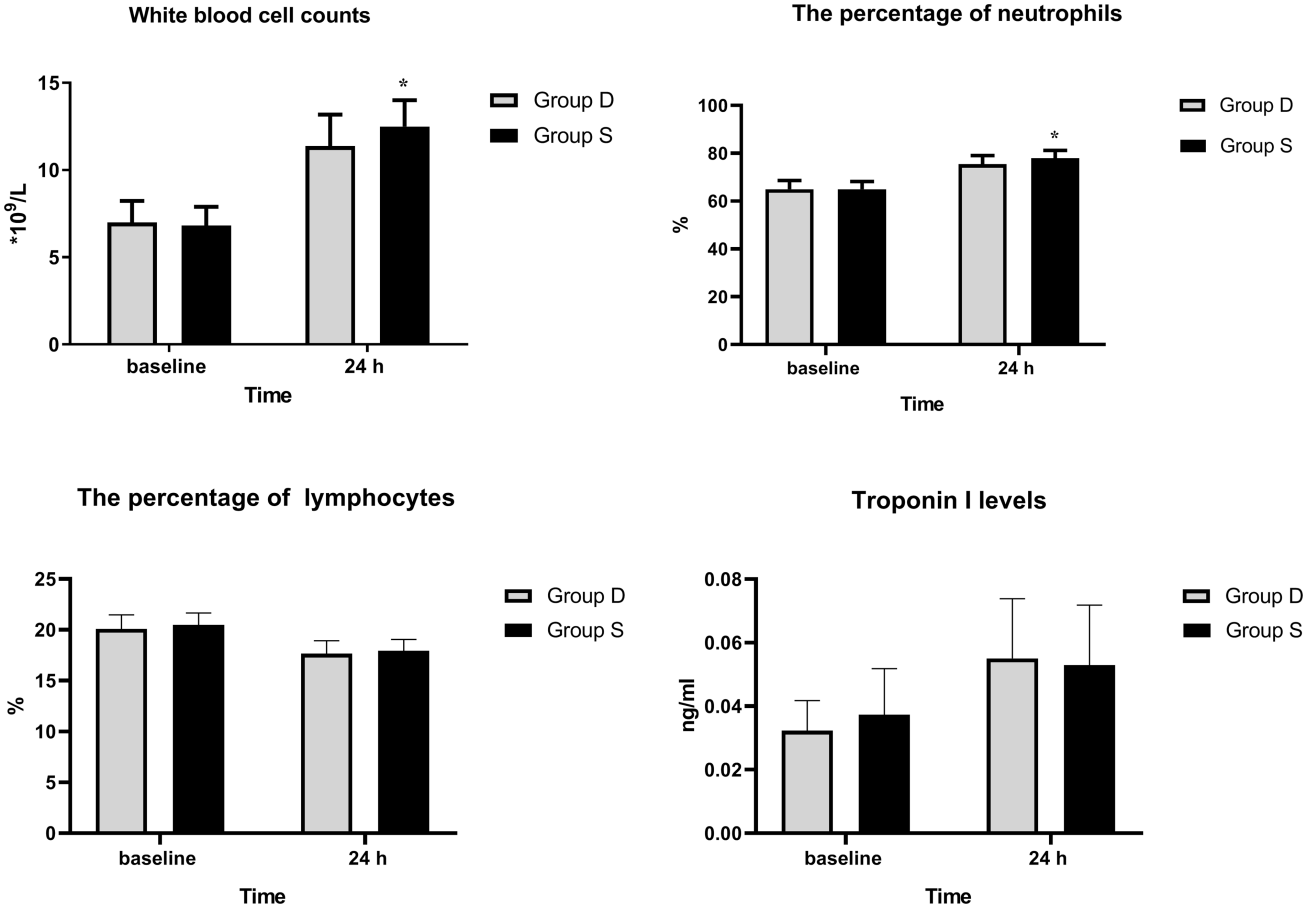
White blood cell counts, percentage of neutrophils and lymphocytes, and troponin I levels preoperatively and 24 h post-surgery.

White blood cell counts and neutrophil percentages decreased significantly 24 h post-surgery in the D group than in the S group (*p* < 0.05; Figure 3). However, lymphocyte percentages and troponin I levels were similar between the groups (*p* > 0.05; Figure 3).

## Discussion

This single-center prospective randomized controlled trial found that the use of desflurane for the maintenance of general anesthesia did not reduce the risk of cognitive impairment compared with sevoflurane. However, desflurane was associated with faster recovery than sevoflurane after general anesthesia in elderly orthopedic patients. Furthermore, patient satisfaction scores and the number of patients willing to undergo surgery with the same sedation regimen were significantly higher in the desflurane group, and white blood cell counts and neutrophil percentages were significantly reduced 24 h post-surgery.

Previous studies have characterized POCD as a condition involving impaired memory and concentration, though its exact pathogenesis remains unclear. The incidence of POCD has been reported to be 25.8% in elderly patients 1 week after surgery and 9.9% 3 months post-surgery (15). It is a multifactorial disorder influenced by patient, surgical, and anesthetic-related factors. General anesthesia, patient predisposition, type of surgery, age, alcohol abuse, low baseline cognition, comorbidities, prolonged preoperative fasting, opioid consumption, intraoperative bleeding, anticholinergic drug load, and excessively deep anesthesia have been considered contribute to the development of POCD (5). In line with previous findings, over 70% of patients in both groups returned to baseline cognitive function within 24 h postoperatively (16).

A previous study has reported that desflurane concentration for maintaining BIS value <50 was 3.58 and 2.75% in middle-aged and elderly patients, respectively, which was lower than that in our study (17). This may be due to the different types of surgeries and anesthesia regimens employed in the two studies. Intraoperative EEG characteristics associated with POCD are more noticeable with sevoflurane than with desflurane (9).

Besides, BIS was lower in patients receiving 1 MAC desflurane than in those receiving 1 MAC sevoflurane, suggesting that provides a deeper level of anesthesia at 1 MAC than sevoflurane (18). However, recent study revealed that intraoperative EEG patterns are associated with POCD development and can be utilized for the early detection of POCD (9). Besides, POCD was less frequent in elderly patients when moderate anesthesia depth was maintained using processed EEG guidance compared with routine care, based on clinical signs and end-tidal anesthetic agent concentration (19). Therefore, in this study, we chose BIS as the monitoring indicator for anesthesia depth and maintained BIS at 40–60 in line with the parameters applied in a previous study (18).

The clinical tools used to measure cognitive function after anesthesia have not been standardized, and the timing of the measurements has varied widely in earlier studies (20). Standard psychometric tests used to measure cognitive ability require a considerable amount of time to administer, which is impractical in the perioperative period. The use of the MMSE to evaluate patients after surgery under general anesthesia has been shown to be both easy and reliable for detecting mild cognitive impairment (21). Moreover, the greater sensitivity of more extensive testing panels is associated with a longer test time, which elderly patients may find challenging to perform in the immediate postoperative period (22). As a result, the MMSE was chosen for use in this study. Different studies have shown a significantly increased risk of developing POCD after desflurane anesthesia in older patients, even though burst suppression duration was shorter under desflurane anesthesia compared to sevoflurane or propofol anesthesia. The main speculation is that the different receptor affinities and induced neurotoxic and inflammatory effects at the cellular level of desflurane may be the underlying cause triggering POCD to a greater extent than propofol or sevoflurane (23, 24). Besides, it decreases CMRO_2_ and preserves cerebrovascular reactivity (25). MMSE scores were lower in the D group at 1 h after surgery compared with those in the S group; however, this difference was not clinically significant. Additionally, over 70% of patients in both groups returned to baseline levels at 24 h postoperatively.

A previous study has reported that the cause of cognitive decline is associated with the inflammation caused by the stress of surgery (26). However, the white blood cell counts and neutrophil percentages were significantly decreased 24 h post-surgery in the desflurane group, although the percentage of lymphocytes and troponin I levels were similar between the two groups. The results were similar to those of a previous study, which found that patients anesthetized with desflurane had significantly better white blood cell counts and percentage of neutrophils than patients in the sevoflurane group. This is likely because volatile anesthesia could significantly enhance both local and systemic oxidative stress, especially with desflurane (27). Furthermore, the systemic stress response caused by the release of cytokines during anesthesia and surgery may cause changes in brain function and play a role in the development of POCD. Neuroinflammation, characterized by inflammatory imbalance and neuronal damage, is an important mechanism that causes neurocognitive disorders during the perioperative period (28).

Inhalational anesthetics are mainly discharged through the respiratory tract and feature convenient administration, easy control of dosage, and stable hemodynamics. The speed of induction and recovery of inhalation agents depends mainly on the blood solubility of each inhalation anesthetic (29). Sevoflurane and desflurane have been widely used and provide relatively rapid induction and emergence due to their low blood solubilities (9). Consistent with the results of a previous study, recovery was significantly faster in the D group. The reason may be that desflurane has the lowest intra-hepatic metabolic rate among the inhalational anesthetics and extremely low toxicity to the liver and kidney. One previous study reported that desflurane increases sympathetic nerve activity, which might also contribute to rapid emergence from general anesthesia (30). Compared with the results of other studies, PACU length of stay was significantly shortened. As a result, the service fees were also reduced, which has a significant economic impact (31). A previous retrospective study indicated that male sex and obstructive respiratory function were factors that contributed to extubation time after general anesthesia with desflurane. In contrast, age, operation time, and BMI were not risk factors for prolonged extubation (32). However, there were no significant differences in patient demographic characteristics between the two groups in this study.

Postoperative respiratory complications are an important quality indicator associated with poor patient outcomes, longer hospital stays, and increased costs. Volatile anesthetics impair hypoxic and hypercarbic ventilatory responses and neuromuscular transmission. They also impair coordination between breathing and swallowing, increasing aspiration risk (33). Consistent with the results of a previous study, desflurane was not associated with reduced postoperative respiratory complications when compared with sevoflurane (34). The postoperative respiratory complications were lower in our study, which aligns with the results of a recent study that reported the use of higher volatile agents is associated with a reduction in risk of postoperative pulmonary complications and mortality compared with patients with total intravenous anesthesia. The reason may be that the immunomodulatory effects of inhalational anesthetics decrease inflammation and attenuate acute lung injury by suppressing inflammatory responses through modulation of alveolar macrophage responses, maintenance of neutrophil recruitment, and GABAA-receptor–mediated anti-inflammatory effects in lung epithelial cells (35).

A previous study reported that desflurane has insurmountable shortcomings such as high cost, and an increased risk of coughing, breath holding, laryngospasm, and tachycardia, especially when the concentrations exceed 1 MAC (36). However, the incidence of emergence cough was significantly reduced compared with the results of other studies. The reason may be partly due to the higher consumption of remifentanil in our study, even though there was no need to adjust the remifentanil Ce to prevent emergence cough between sevoflurane and desflurane anesthesia in elderly female patients (37). However, no increase in the incidence of adverse reactions such as respiratory depression, delayed emergence, nausea, and vomiting was recorded. A previous study reported that volatile anesthetics were potent greenhouse gases and a major contributor to environmental waste generated from the operating room, especially desflurane, which remains in the atmosphere for nearly 14 years and is known to have a global warming potential (GWP) that is 20-fold greater than sevoflurane (38). All of these defects limit the clinical application of desflurane. Moreover, volatile anesthetics can increase post-extubation shivering compared to propofol-based anesthesia, and some show a dose-dependent increase (39). In summary, clinical efficiency must be balanced with environmental responsibility. As stated in the previous investigation, the shift in practice by anaesthetists away from anaesthetic gases with high global warming potential toward lower emission techniques (e.g., total intravenous anaesthesia) could result in significant carbon savings for the health system (40).

This study has several limitations. First, in addition to MMSE, more sensitive tools such as the Saskatoon Delirium Checklist, Digit-Symbol Substitution Test, and Geriatric Mental State Examination have all been used to evaluate cognitive function in elderly patients. These tests assess recovery of consciousness, perception, orientation, coherence, memory, and motor activity (1). The use of a more sensitive psychological test of cognitive dysfunction associated with a longer test time might have demonstrated more prolonged impairment of cognitive performance after surgery and strengthen the clinical interpretation of the cognitive data. Second, the results of adverse reactions in this study were not specifically powered, and a large-volume study is needed to evaluate these values. Third, this study design specifies a single-center trial involving patients aged 65 to 75 years from one hospital. Such restriction limits the representativeness of the population. Future multicenter trials or inclusion of a broader age range and diverse ethnic or clinical backgrounds are needed to enhance the external validity of the findings. Fourth, Considering the small number of patients included in this study and in order to reduce statistical bias, we did not perform a *post hoc* subgroup analysis by sex though physiological responses to anesthetics may differ between men and women. Besides, multivariate regression or ANCOVA models are needed to verify whether the observed differences between anesthetics remain significant after adjusting other clinical covariates such as surgery duration, comorbidities, opioid dose, or intraoperative hemodynamics. Finally, we only performed cognitive testing at baseline, 1, 6, 24, and 48 h post-surgery, which could have missed cases within 48 h post-surgery due to the present time points or late cases beyond 48 h.

In conclusion, desflurane was not associated with reduced MMSE scores or postoperative respiratory complications, though recovery was significantly faster, and patient satisfaction scores were higher compared with sevoflurane. As a result, we should adhere to individualized treatment based on the advantages and disadvantages of each drug in clinical applications. Further research is needed to explore the relationship between anesthetic selection and long-term cognitive or economic outcomes.

## Data Availability

The original contributions presented in the study are included in the article/supplementary material, further inquiries can be directed to the corresponding author.
